# Supplementary material for: Morphological and Molecular Characterization of Calonectria foliicola Associated with Leaf Blight on Rubber Tree (Hevea brasiliensis) in Thailand
Source: J Fungi (Basel). 2022 Sep 20;8(10):986. doi: 10.3390/jof8100986 (PMC9604915; doi:10.3390/jof8100986)
Supplement: Supplementary file 1 [file jof-08-00986-s001.zip › jof-1889620-supplementary.pdf]

Table S1. Sequences from GenBank used in phylogenetic tree analysis.

Species	Isolate	Location	Substrate	GenBank Accession number			
				**ITS**	***tub2***	***cal***	***tef1-α***
Calonectria auriculiformis	CBS 143561^T = CMW 47178	Hau Loc, Thanh Hoa, Vietnam	Soil in *Acacia auriculiformis* plantation	MT359651	MH119287	MH119254	MH119221
	CBS 143562 = CMW 47179	Hau Loc, Thanh Hoa, Vietnam	Soil in *Acacia auriculiformis* plantation	MT359652	MH119288	MH119255	MH119222
Ca. brasiliensis	CBS 230.51^T	Aracruz nursery, Brazil	Leaves of *Eucalyptus* sp.	MT359661	GQ267241	MT335200	GQ267328
	CBS 114257	Aracruz nursery, Brazil	Leaves of *Eucalyptus* sp.	GQ280625	GQ267242	GQ267422	GQ267329
Ca. candelabra	CPC 1675	-	-	-	FJ972426	GQ267367	FJ972525
	CMW 31001 = CSF11405	-, Brazil	*Eucalyptus* sp.	MT359669	GQ421779	MT335208	-
Ca. cerciana	CBS 123693^T=CMW 25309	Guangdong Province, China	*Eucalyptus urophylla* × *E. grandis*	MH863319	FJ918510	GQ267369	FJ918559
	CBS 123695=CMW 25290	Guangdong Province, China	*Eucalyptus urophylla* × *E. grandis*	MT359673	FJ918511	GQ267370	FJ918560
Ca. cylindrospora	CBS 110666	-, USA	-	GQ280626	FJ918509	GQ267423	FJ918557
	CBS 119670	nursery Universita degli Studi di Catania, Italy	Symptomatic leaf and stem tissues of *Pistacia lentiscus*	KY653251	DQ521600	MT335236	GQ421797
Ca. foliicola	CBS 136641 = CERC 1728 = CMW 31393 = CPC 23491	Guangxi Province, China	*Eucalyptus urophylla* x *E. grandis* clone	-	KJ462916	KJ463031	KJ462800
	CERC 1729 = CMW 31394 = CPC 23492	Guangxi Province, China	*Eucalyptus urophylla* x *E. grandis* clone	-	KJ462917	KJ463032	KJ462801
	CERC 1730 = CMW 31395	Guangxi Province, China	*Eucalyptus urophylla* x *E. grandis* clone	MT359709	KJ462918	KJ463033	KJ462802
	Calokb001	Krabi Province, Thailand	*Hevea brasiliensis* leaf	**LC721579**	**LC721587**	**LC721575**	**LC721583**
	Calokb002	Krabi Province, Thailand	*Hevea brasiliensis* leaf	**LC721580**	**LC721588**	**LC721576**	**LC721584**
	Calokb003	Krabi Province, Thailand	*Hevea brasiliensis* leaf	**LC721581**	**LC721589**	**LC721577**	**LC721585**
	Calokb004	Krabi Province, Thailand	*Hevea brasiliensis* leaf	**LC721582**	**LC721590**	**LC721578**	**LC721586**
Ca. hodgesii	CBS 133609^T	Minas Gerais, Vicosa, Brazil	Root of *Anadenanthera peregrina* seedlings	-	KC491228	KC491222	KC491225
	CBS 133608	-, Brazil	Stem of *Piptadenia gonoacantha*	-	KC491227	KC491221	KC491224
	CBS 133610	-, Brazil	Leaf of *Azadirachta indica*	-	KC491229	KC491223	KC491226
Ca. insularis	CBS 114558^T	Tamatava, Madagascar	Soil	GQ280587	-	GQ267389	FJ918556

	CBS 114559	Tamatava, Madagascar	Soil	GQ280588	-	GQ267390	FJ918555
Ca. maranhensis	CBS 134811[T]	Açailândia, Brazil	Eucalyptus leaf	-	KM395948	KM396035	KM395861
	CBS 134829	Urbano Santos, Brazil	Soil	-	KM395952	KM396039	KM395865
Ca. papillata	CBS 136097[T] = CPC 23517	Guangdong Province, China	Soil	KY653267	KJ462964	KJ463079	KJ462849
	CBS 136084	Guangdong Province, China	Soil in Eucalyptus plantation	-	KJ462962	KJ463077	KJ462847
	CBS 136096	Guangdong Province, China	Soil in Eucalyptus plantation	KY653266	KJ462963	KJ463078	KJ462848
	CBS 136251	Guangdong Province, China	Soil in Eucalyptus plantation	-	KJ462965	KJ463080	KJ462850
Ca. polizzii	CBS 123402[T] = CMW 7804	Sicily, Carrubba, Italy	*Arbutus unedo*	GQ280666	FJ972417	GQ267461	FJ972486
Ca. propaginicola	CBS 134815[T]	Santana, Brazil	Eucalyptus	-	KM395953	KM396040	KM395866
	CBS 134820	Santana, Paŕa, Brazil	Used planting substrate	-	KM395956	KM396043	KM395869
	CBS 134821	Santana, Paŕa, Brazil	Used planting substrate	-	KM395957	KM396044	KM395870
Ca. pseudocerciana	CBS 134824	Santana, Paŕa, Brazil	Eucalyptus seedling	-	KM395962	KM396049	KM395875
Ca. pseudohodgesii	CBS 134818	Minas Gerais state, Viçosa, Brazil	*Azadirachta indica*	-	KM395903	KM395989	KM395815
	CBS 134819	Minas Gerais state, Viçosa, Brazil	*Azadirachta indica*	-	KM395906	KM395992	KM395818
Ca. sulawesiensis	CBS 125277[T] = CMW 14878	Sulawesi, Indonesia	Leaves of *Eucalyptus* sp.	GQ280635	GQ267220	GQ267432	GQ267340
	CBS 125253 = CMW 14879	Sulawesi, Indonesia	Leaves of *Eucalyptus* sp.	GQ280637	GQ267222	GQ267434	GQ267342
Ca. terrestris	CBS 136642[T] = CERC 1856 = CMW 35180	Guangdong Province, China	Soil	-	KJ463004	KJ463121	KJ462891
	CBS 136643 = CERC 1868 = CMW 35364 = CPC 23493	Guangdong Province, China	Soil	-	KJ463005	KJ463122	KJ462892
	CBS 136644 = CERC 1870 = CMW 35366 = CPC 23494	Guangdong Province, China	Soil	-	KJ463006	KJ463123	KJ462893
	CBS 136645 = CERC 1854 = CMW 35178 = CPC 23496	Guangdong Province, China	Soil	-	KJ463007	KJ463124	KJ462894
	CBS 136651 = CERC 1937 = CMW 37974 = CPC 23516	Guangdong Province, China	Soil	-	KJ463009	KJ463126	KJ462896

Ca. tetraramosa	CBS 136635^{T} = CERC 1809 = CMW 31474 = CPC 23489	Guangdong Province, China	*Eucalyptus urophylla* x *E. grandis* clone	KY653282	KJ463011	KJ463128	KJ462898
	CBS 136637	Guangdong Province, China	*Eucalyptus urophylla* x *E. grandis* clone	-	KJ463012	KJ463129	KJ462899
Curvicladiella cignea	CBS 101411	-, French Guiana	Decaying seed	KM231744	KM232001	KM231285	KM231866
	CBS 109167^{T}	-, French Guiana	Leaf litter	-	KM232002	KM231287	KM231867

T=Ex-type strain, Isolates obtained in this study are indicated in **bold**, - : Not available
